# A nature-positive future with biological invasions: theory, decision support and research needs

**DOI:** 10.1098/rstb.2023.0014

**Published:** 2024-05-27

**Authors:** Melodie A. McGeoch, David A. Clarke, Ninad Avinash Mungi, Alejandro Ordonez

**Affiliations:** ^1^ Securing Antarctica's Environmental Future, School of Biological Sciences, Monash University, Clayton 3800, Victoria, Australia; ^2^ Section of Ecoinformatics and Biodiversity, Department of Biology, Aarhus University, Aarhus 8000, Denmark; ^3^ Center for Ecological Dynamics in a Novel Biosphere (ECONOVO), Department of Biology, Aarhus University, Aarhus 8000, Denmark

**Keywords:** global biodiversity framework, community ecology, novel ecosystems, adaptive management, prioritization, species movement

## Abstract

In 2050, most areas of biodiversity significance will be heavily influenced by multiple drivers of environmental change. This includes overlap with the introduced ranges of many alien species that negatively impact biodiversity. With the decline in biodiversity and increase in all forms of global change, the need to envision the desired qualities of natural systems in the Anthropocene is growing, as is the need to actively maintain their natural values. Here, we draw on community ecology and invasion biology to (i) better understand trajectories of change in communities with a mix of native and alien populations, and (ii) to frame approaches to the stewardship of these mixed-species communities. We provide a set of premises and actions upon which a nature-positive future with biological invasions (NPF-BI) could be based, and a decision framework for dealing with uncertain species movements under climate change. A series of alternative management approaches become apparent when framed by scale-sensitive, spatially explicit, context relevant and risk-consequence considerations. Evidence of the properties of mixed-species communities together with predictive frameworks for the relative importance of the ecological processes at play provide actionable pathways to a NPF in which the reality of mixed-species communities are accommodated and managed.

This article is part of the theme issue ‘Ecological novelty and planetary stewardship: biodiversity dynamics in a transforming biosphere’.

## 1. Rationale for a nature-positive future with invasive alien species

A ‘nature-positive future’ (NPF) is an aspirational vision of nature in the future, painted for the purpose of setting trajectories for how we might achieve outcomes that are positive for nature and society [1,2] (see table 1; glossary). In practical terms, the NPF vision accepts that biodiversity losses are inevitable. However, they should be more than fully compensated so that nature is retained or restored in net terms [1]. The key phrase is 'net terms’, which implies a balance between achieving human development and preventing biodiversity losses that are not ecologically replaceable or socially acceptable [6] (table 1; glossary). The shift in thinking and emphasis that NPF with biological invasions (NPF-BI) brings is an acceptance that the living world is already irreversibly changed, in most cases owing to long trajectories of human land use and the movement of plants and animals across biogeographical barriers. Consequently, what we envisage for biodiversity and future ecosystems demands a clear vision of how to ensure measurable maintenance or net gains in biodiversity. These ideas are being promoted for uptake in multinational biodiversity policy [1,6], and are already formalized as emerging from biodiversity offsetting in some national planning and development agendas [3,8].

**Table 1.** Glossary of concepts and their meanings as used in this paper.

concept	meaning
alien species^1^	synonymous with ‘introduced species’
biodiversity net gain	an overall inrease in biodiversity in an area over a given period of time. Measuring net biodiversity gain is complicated by the multiple ways in which biodiversity must be measured for a comprehensive assessment thereof. The term originates from the conservation field of biodiversity offsetting [1,3–5]
invasive alien species (IAS)^1^	a subset of introduced species that have negative impacts
introduced species^1^	synonymous with ‘alien species’. Used here to denote species that are introduced to a new locality by human agency, with populations that are established in the new locality. When an introduced species starts to have negative impacts it is referred to as an ‘invasive alien species (IAS)’, i.e. the concepts of introduced and invasive are hierarchical
mixed-species community	used here to refer to a community of species consisting of a mix of populations of both native and introduced species, and where the number of introduced species present can range from one to several
multispecies invasion	the persistence of multiple alien species as part of natural and semi-natural landscapes forming mixed-species communities
nature-positive future (NPF)	to halt and reverse nature loss measured from a baseline of 2020, through increasing the health, abundance, diversity and resilience of species, populations and ecosystems so that by 2030 nature is visibly and measurably on the path of recovery (naturepositive.org)
	here we use the term nature-positive future in its strictest sense, i.e. explicitly the requirement for a measurable net increase in biodiversity. In this sense it is equivalent to ‘biodiversity net gain’ [2,6]
nature-positive future with biological invasions (NPF-BI)	referring exclusively to the later stages of the invasion process when introduced species have become persistent members of native communities or transformed landscapes, i.e. where prevention and eradication have failed. We argue that the theory of community ecology has a critical role to play in understanding the dynamics of mixed-species communities, and in adequately estimating biodiversity net gain (here measured as native richness, native abundance and/or ecosystem function) at management-relevant scales appropriate to achieve NPF-BI

^1^For internationally accepted definitions in invasion biology, with which the interpretations above are consistent, see IPBES [7].

How do biological invasions and their consequences fit within this thinking? Most of the world's terrestrial and freshwater ecosystems are currently home to many introduced species [9,10]. By 2050 and beyond, these species mixes (native and human-introduced) will probably persist into the future, in some cases with new introductions and ongoing range-infilling and local adaptation and evolution by extant introduced species. With mixed-species communities (see table 1; glossary) as a long-term reality, what implications do these species mixes have for the sustainable management of landscapes? Furthermore, how can we incorporate these changes into the vision of a NPF-BI? Although the idea of NPF-BI is new and little considered in a biological invasions context to date, it parallels recent advances to strengthen the connections between ecological theory (particularly community ecology) and invasion biology [11,12] as a basis for understanding the long-term consequences of biological invasion. While very different, both these developments recognize and emphasize that invasive alien species  (IAS) are inherently part of, and intricately connected to, the areas and communities that they successfully invade. Therefore, envisioning how the interactions and dynamics of invaded systems play themselves out in the long-term, and how such thinking may influence management decisions now and in the future, is necessary.

Introduced species either subtract from (e.g. driving native population and species extinctions [13]) or add to a net change in biodiversity (e.g. enhancing local richness or providing habitat for threatened species [14,15]). Similarly, they can degrade or enhance societal values such as human health and resource provision [16]. Therefore, central to the intent and ambition of a NPF is the active management of mixed-species communities to support positive effects and avoid negative ones, recognizing the overwhelming dominance of evidence of negative impacts of IAS on people and nature [17]. Here, we provide a long-term perspective on the stewardship of biodiversity encompassing this persistent form of global change, based on an overview of the current understanding of multispecies invasion (table 1; glossary). We focus principally on the post-introduction and establishment stages of the invasion process, acknowledging that prevention of new introductions will remain an essential precaution given the inevitable uncertainty associated with the dynamics of biological systems. From a NPF-BI perspective, the goals and approaches used to manage future landscapes will require explicit consideration of the populations of introduced species within them.

## 2. Insights for a nature-positive future with biological invasions from 70 years of invasion biology

The process of human-induced biological invasion is very well understood [7,18,19]. This body of knowledge provides a foundation for the premises upon which NPF-BI might be based and the broad approaches that could be taken to shift to such a future (table 2).

**Table 2.** Managing mixed species communities to achieve a nature-positive future (NPF) requires careful consideration of the lessons learned from 70- years of invasion ecology. Here we list key premises for a NPF with biological invasions (NPF-BI), based on this foundation, and associated with actions linking them to the goals of NPF-BI.

NPF-BI premise	response/action for NPF-BI
species populations: it is more ecologically informative to consider invasive alien populations rather than invasive alien species (IAS) at landscape scales, because benign populations of the same species exist (in the introduced range) and in most cases out-number those causing local harm	the management goal for particular introduced species populations is determined by the effect they have on net biodiversity and ecosystem outcomes in a landscape context, including consideration of climate change impacts and including the multiple considerations for dispersal management (figures 1 and 2). Context-relevant management of populations of introduced species is imperative to avoid net biodiversity losses
landscape scales: the most relevant scale for a NPF-BI is the landscape—encompassing communities and metacommunities—because this is the scale at which impacts are incurred and environmental values are managed	improve understanding of biodiversity and ecosystem change and management alternatives at this focal scale, while considering influential processes and their temporal dimensions beyond and within the landscape scale
temporal dimensions: time and a dynamic continuum of invasion is a fundamental property of the invasion process and its relationship with biodiversity and ecosystem change across landscapes	identifying the most successful invasion management strategy for NPF-BI requires actions that consider the temporal development of community invasion and that are appropriately designed for the stage and level of community invasion
ecological processes: once an introduced species propagule arrives in a landscape, it faces the same mechanistic opportunities and constraints faced by native propagules dispersing in the metacommunity	managing landscapes for NPF-BI include modulating and manipulating the processes by which introduced and native species operate (dispersal, drift, environmental filtering, within and across trophic level interactions and genetic change)
trajectories of change within phase space: managing communities along trajectories of change within a state space defined by invasion level, dominance and functional change can deliver net positive outcomes for biodiversity and ecosystems	management decision frameworks and support tools, such as the resist-accept-direct (RAD) continuum help to guide the desirability and feasibility of three broadly different management goals for landscapes. In the context of NPF-BI, the RAD approach provides a framework to promote biodiversity or ecosystem gains by resisting or directing change, and the losses incurred from accepting them
functional traits: the dominance of individual and multiple introduced species depends on traits that provide them with an ecological advantage rather than on their provenance. Locally realized structural and functional outcomes of multispecies invasion can differ in magnitude and direction from general or averaged comparisons of native and introduced species traits	mitigating and preventing the possible negative effects of introduced species requires considering the direct and indirect factors conferring ecological benefits, as well as understanding community-wide trait changes
feedbacks and novel interactions: changes in negative feedbacks (e.g. density dependence, parasitism, herbivory, predation) and positive feedbacks (i.e. Invasion meltdown) affect the success and impacts of populations of introduced species	interactions and feedbacks can be disrupted or restored to reduce the negative impact of introductions on native communities. New interactions in mixed species communities can in some instances contribute to NPF-BI by replacing lost functions, by delivering valuable ecosystem services, or supporting elements of native biodiversity in transformed and transforming communities
emergent properties and ecosystem function: while IAS are drivers of ecological novelty and changes in ecosystem functions, it is less clear how multispecies invasions affect ecosystem function	a fundamental knowledge gap to fill in the near future is the impact of multispecies invasions on ecosystem functions and the services they provide

Recent taxon-specific and cross-taxon collations of IAS data at a global scale have revealed that more than 23 000 introduced species are of environmental concern [20]. Ecological impact has, however, been recorded in only 16% of these cases (across country level occurrences of IAS, including 101 000 country-by-species records), with more than 50% of these species of concern recorded in single countries only [20]. One of the hallmarks of invasion success is this context dependence, with increases in the abundance, spread and impact of particular populations of introduced species far more successful in some instances than in others [21–24]. For example, biodiversity impacts by the successful, widespread and comparatively well-studied invasive alien Argentine ant (*Linepitheme humile*) show clear context-dependence, with strong effects detected on some taxonomic groups and in some localities and little to no effect detected in others [25]. In some instances the reasons for such context dependence remain unknown, but in others landscape-level abiotic heterogeneity is clearly responsible (e.g. disturbance, local climate, soil conditions) [26,27]. The context dependence of invasion suggests that it may be more ecologically informative to refer to invasive alien populations rather than IAS, acknowledging that benign (net neutral) populations of the same species may exist and outnumber those causing local harm. This is a key premise of NPF-BI (table 2).

Regardless of the generally low proportion of populations of introduced species that go on to have substantive environmental impacts, those that do can bring about significant biodiversity loss, irreversible changes to ecosystem function, and substantial socio-economic harm. The effect size of ecological changes brought about by IAS significantly outweighs other global change drivers (e.g. drought, warming, nitrogen deposition), with the local ecological impacts wrought by invasives on their own being as large as changes resulting from interactions between different drivers of change [28].

Once populations of newly introduced species have established and started to spread, often it becomes impossible or undesirable to eradicate them, as they become persistent elements of local communities and may even begin to evolve *in situ* [29,30]. Once entrenched, populations of introduced species become one of multiple factors to consider when managing landscapes for their biodiversity, ecosystem and societal values [30]. These can include, for example, the management of fire, water flows and human–wildlife interactions, alongside unprecedented weather events [31]. Climate change adds to the uncertainty of local scale dynamics of multispecies invasions [32,33], and the multiple and interacting drivers of ecological change further contribute to the high context dependence of realised outcomes at local scales [22,27,28]. Therefore, deliberate invasion management (including decisions for no action) becomes a fundamental part of broader environmental stewardship to achieve a NPF-BI, where considering multiple and interacting drivers of change is the norm [34].

While we identify the landscape scale as the focal unit for understanding and management, and communities and metacommunities as the concomitant level of organization, scale considerations remain central to both research and management for NPF-BI (table 2). There is pervasive evidence for the scale-dependence of pattern and process in ecology broadly, but also in invasion ecology specifically [35]. Scale dependence is evident when detecting the effects of environmental filtering, competitive interactions [36], phylogenetic relatedness [37], and the relationship between species richness of native communities and invasion success [22]. The invasion process is also by definition a temporal one, including ongoing propagule pressure from the regional species pool. Time since invasion and community successional stages are therefore key considerations in decisions about invasion management ([35]; table 2).

## 3. The ecology of mixed species landscapes under a nature-positive future with biological invasions

### (a) Theory

Community ecology is a field of scientific endeavour with high uncertainty and struggles to achieve generality, addressing as it does the many ways in which multiple species interact under a range of environmental contexts [38,39]. It is in this already dynamic context that alien species arrive, establish, interact and, in some cases, go on to have effects that detract from net positive outcomes for biodiversity and ecosystems. Nonetheless, contemporary community and metacommunity ecology provides well-established general principles and mechanistic frameworks at scales appropriate for understanding mixed-species communities and metacommunities [36,40].

A synthesis of hypotheses in invasion biology [11] provides a foundation for the need to manage mixed-species landscapes to achieve nature-positive outcomes. Three clusters of hypotheses in invasion biology together capture the importance of local abiotic conditions and resource availability (environmental filtering), species traits (including evolutionary genetic potential) and biotic interactions (within and between trophic levels) [11]. Alongside propagule dynamics and dispersal, as well as the history of local communities, these mechanisms proposed to explain the success of introduced species in native communities have strong parallels with the processes of community assembly and maintenance [12,40]. A synthesis of hypotheses and theory across community ecology and invasion biology identified the same six processes used to explain native community assembly and dynamics and the post-introduction invasion process, i.e. dispersal, drift, abiotic environmental filtering, within and across trophic level interactions and genetic change [12]. In other words, once a human-introduced propagule arrives in a landscape, it faces the same theoretical mechanistic opportunities and constraints faced by native propagules dispersing in the metacommunity (table 2).

However, the relative focus across domains differs, and invasion biology has yet to fully embrace research on the full suite of processes at play in the assembly and maintenance of mixed-species communities [12]. More generally, while hypotheses in invasion biology have strong parallels with those in community ecology, support for many of these are either taxonomically biased (for example, plants versus animals); dominated by population level, single and two-species studies; come from relatively few studies; and/or support across taxa and systems is mixed [36,41]. Managing ecological processes, for example preventing or facilitating propagule dispersal is already a well-established action in conservation management [42]. Understanding the ecological processes in invaded landscapes and mixed species communities [12], and the relative importance of these processes in driving change along multi-species invasion trajectories [36,41], will help to predict how the process could unfold. It should also identify mechanisms that could be manipulated to influence or change this trajectory, i.e. offering potential management solutions [43].

### (b) Quantifying the structure and function of mixed species communities

**Figure 1.**
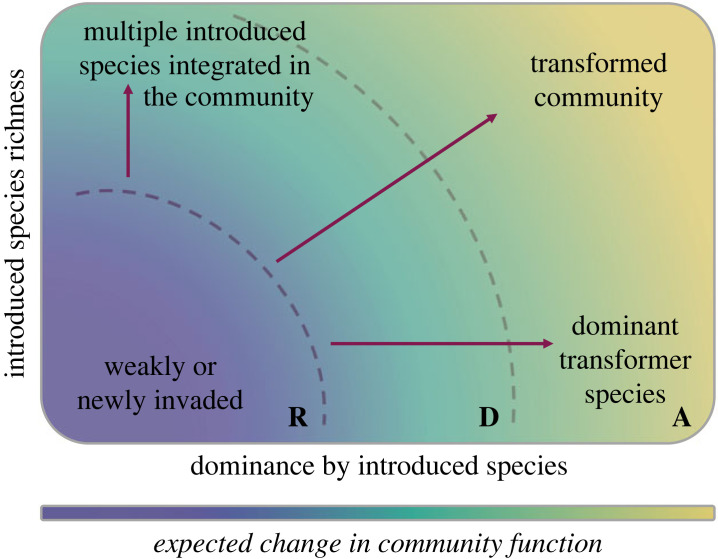
Alternative futures for mixed-species landscapes. Communities of species that include a mixture of introduced and native taxa lie on a continuum along three axes, from low (bottom left) to high (top right) levels of invasion. Landscapes are characterized by the numbers or proportions of introduced species (richness), different relative dominance (measured as abundance, cover or biomass) of these introduced species, either individually or collectively, and measured or expected change to emergent ecological functions as a consequence of species identity and dominance changes (colour shading). Time is an inherent dimension in the representation of change across this three-dimensional space. Nature-positive future management for biodiversity would aim to maintain or shift the community to the lower left of the community phase space. Management for ecosystem function will be determined by the species traits of the alien species, how these differ from native species in the community, and collectively how these drive changes in emergent ecosystem processes and functions. A, resist; B, direct; C, accept—broadly aligned with the colour gradient (see text).

Research on the community-level consequences and management of multispecies plant invasion is growing (e.g. [26,36,44]), and more studies of mixed-species animal communities have been called for [41]. In practice, information on the structure and function of mixed species communities serves a different and complementary purpose to understanding the ecological processes outlined above. Under NPF-BI, being able to place a community along the multidimensional community invasion trajectory in figure 1, with data on structure and function, will guide the management goals for that community. For example, multiple comparatively rare alien species in the landscape might trigger an intensive, multispecies eradication effort. By contrast, multiple dominant introduced populations would guide management towards focusing on protecting priority threatened species or managing particular ecosystem services.

The structure and function of mixed-species communities can be characterized by the relative numbers of native and alien species, the relative dominance (abundance, cover or biomass) by one or more alien species, and the emergent changes to ecosystem functions that result from these community-wide properties (figure 1). Based on these conditions, future landscapes can be considered as lying along a multidimensional invasion continuum, bounded by one to several alien species that individually and jointly constitute a low to high relative abundance in the community, and any net change in emergent function that occurs as the community change process unfolds (figure 1). Variation in the relative prevalence of all the key processes outlined above, over both ecological and evolutionary time scales, drives the transition of communities through the phase space of figure 1. A concerted effort to quantify and understand the patterns and processes of mixed species communities is a research priority for setting trajectories for management under NPF-BI (table 2).

### (c) Current understanding of mixed species communities

The current understanding of mixed-species communities relevant to NPF-BI is incomplete but is starting to coalesce across three inter-related areas.

#### (i) Species origin, identity and functional traits

A growing body of work shows that species origin (as native or introduced) can be less important than the characteristics of a species' population dynamics (rare or common, newly colonized or established) or trait combinations [45–47]. For example, common plant species respond similarly to nutrient addition and intraspecific competition, regardless of their origin as native or introduced [48–50]. For some alien species, trait and context combinations do have predictive power for how the species is likely to interact in a recipient community. For example, social insects have multiple traits that render them disproportionately successful amongst insect invaders not only in establishment and spread, but also often through competitive interactions with native taxa [51,52]. Similarly, nitrogen-fixing plant invasions of oligotrophic ecosystems, such as invasive *Acacia*, trigger persistent and significant changes in soil quality and native plant communities [53,54]. Performance (measured as growth or carbon capture) tends to be higher for alien species [55,56] as they tend to be located on the high end of ecological strategies related to performance (e.g. along the leaf economics spectrum) [57].

The mix of traits of multiple alien species in communities affects the outcomes for community structure and function and, therefore, the biodiversity and ecosystem consequences of multispecies invasion [41]. For example, alien species in mixed-species animal communities can have superior performance for several traits compared to native community members (such as growth rates, physiological tolerance or higher fecundity), although such animal community-level studies are rare [41]. In general, introduced and native plants are phylogenetically similar and their traits are functionally distinct when contrasted with co-occurring natives [58], although at local, *in situ* community to landscape scales, these patterns can vary and be scale-dependent [44]. In plant communities, community-wide traits such as seed mass, specific leaf area and herbivore resistance have been shown to be correlated with invasion success at particular spatial scales and points of time in the invasion process [21,59]. The predictive power of these results across all invasions may however be undermined by a bias towards research on the most damaging alien species [60]. The interactions between suits of realised traits at community scales, and how these shift communities across species richness, dominance and emergent function, is of central consequence for NPF-BI (table 2).

#### (ii) Feedbacks and interactions

Feedbacks across ecological processes set trajectories of change that need to be either disrupted or facilitated to maintain net positive biodiversity and ecosystem outcomes. The dominance of negative feedbacks from intraspecific interactions (density dependence), for example, drive and maintain species rich communities, whereas positive feedbacks (such as facilitation, biotic-abiotic interactions) can drive the composition of communities towards single species dominants [61]. Invasion-driven meltdown may occur when introduced species alter the local biotic or abiotic environment in ways that start to exclude native community members. However, in both plant and animal communities, species richness can mediate the likelihood of directional invasion resulting from invader impacts on abiotic conditions (such as plant-soil feedbacks) [62]. For example in a sessile epibenthic fouling community, space availability and species richness (regardless of species origin) drove introduced species abundance, showing that trajectories to invasion meltdown are not inevitable [63]. Cases of invasion meltdown are in fact comparatively few, but community change of various degrees towards this, albeit not inevitable outcome, may be more prevalent. Indeed, some balance of positive and negative feedbacks, along with dispersal, drift and genetic change, may maintain mixed-species communities well-within the bounds of community phase space [61,64]. Significant evidence exists for the role of abiotic conditions (that drive environmental filtering and population performance), as well as abiotic heterogeneity (such as disturbance regimes and soil quality) as key determinants of invasion dynamics [65,66], again pointing to the relevance of landscape and finer scales to invasion outcomes.

In some cases, novel interactions contribute to net positive outcomes for biodiversity and ecosystems [14,67,68]. For example, with seed dispersers in global decline limiting the capacity of plant species to track changing climates, in some instances alien bird species are facilitating long-distance seed dispersal [69]. Similarly, introduced large herbivores may play a role as novel analogues of herbivores now long extinct, and reinstate lost ecological functions (e.g. [70]). Some alien populations deliver provisioning services that slow biodiversity loss and mitigate the impacts of global change [15]. For example, many Northern Hemisphere ecosystems depend on reinstating the functional analogue of lost megafauna, including species sourced and rewilded from semi-wild populations (e.g. European bison and cattle in rewilding Europe). Novel megafaunas are considered to increase biodiversity, ecological function, and maintain physical processes such as nutrient transfer ([71]; table 2).

#### (iii) Emergent properties and ecosystem function

When the increase in richness and/or dominance of introduced species results in irreversible structural changes, emergent ecosystem function may also be substantially different (so-called ‘novel ecosystems’ [58,72]). Alternatively, introduced aliens can provide similar functions to their native counterparts with little substantive change in function at a community level [73,74]. Largely single species studies show that invaders can alter the amount, distribution, and the flux of biochemical pools and nutrient inputs of ecosystems, but again these outcomes are context dependent [75]. One of the most general correlations between change in a community property and a shift in ecosystem processes relates to the relative abundance, biomass and dominance of introduced species [76]. High alien biomass can, for example, drive the accumulation of herbivores via an increase in community-wide specific leaf area characteristic of these species, which in turn increases decomposition rates ([67]; table 2).

The specific impacts of particular introduced species on ecosystems have been described and quantified using a range of methods [13]. The impacts of multispecies invasions on ecosystem function requires either the direct measurement of whole system biochemical budgets [75] or an examination of experimental and *in situ*, community-wide trait properties associated with functions of interest [37,44,67]. Current understanding shows that relationships between introduced species richness, traits and emergent ecosystem properties are non-linear [75], and likely to vary along continua within multidimensional phase space (for example within the three dimensions of figure 1). Focusing on the nature of these continuous, potentially multidimensional trajectories of change in the structure of communities, and how these trajectories drive emergent functions, can deliver the understanding needed to manage landscapes for NPF-BI (table 2).

## 4. Responding to a future committed to mixed species landscapes

Determining the appropriate stewardship approach for a mixed-species landscape to achieve NPF-BI requires assessing where a particular community lies along the continuum of biological configuration changes and functional shifts (figure 1), and how this position aligns with local priorities for the conservation of biodiversity and ecosystems [30,77]. NPF-BI accepts that, in most landscapes, this trajectory of change has been initiated and that management goals for individual landscapes (communities) will determine how far it has and will advance along the trajectory. The stewardship of mixed species landscapes of the future to achieve a NPF-BI will be determined by what biodiversity and ecosystem values are desired, the feasibility of maintaining or restoring them, and the societal transformations necessary to achieve this goal, all topics beyond the scope of this review.

Decision-making needs to be informed by a prioritization process that identifies where the largest contributions to NPF-BI can be achieved, along with identifying the overarching management objective/goal for priority landscapes [78]. For example, 66% of India's natural areas are invaded making it impossible to manage the full extent of invaded area for invasions [79]. Management activities (eradication, control and restoration) are therefore prioritized in areas with guaranteed biodiversity returns [79]. For example, the ‘restorative continuum’ from reducing societal impact, through to restoring ecological function (rehabilitation) to recovering native ecosystems (restoration) provides clear direction for the appropriate setting of stewardship goals to achieve a NPF-BI across a range of contexts [77]. Decision-support frameworks are emerging as useful for guiding stewardship of landscapes along change continua, and we discuss two particularly relevant to NPF-BI.

### (a) When and how to intervene to achieve net positive outcomes

Managing the variability, dynamics and uncertainty of community trajectories and emergent functioning of landscapes and their values, demands ongoing evaluation and intervention and an appreciation of the continuous nature of biodiversity change [80]. A management framework that embodies such a continuum of trajectories of change (figure 1) is the resist–accept–direct (RAD) framework [74,81]. In a NPF-BI context, the RAD framework can be interpreted as: resist (R) as the conventional approach to prevent, eradicate and control negative changes following biological invasions for the purpose of maximizing biodiversity value; direct (D) as accommodating persistent alien species that have a neutral or desirable effect on the ecosystem or its value, including interventions to ‘direct’ mixed species communities towards more desirable, resilient and biodiverse states; and accept (A) as interventions that ‘accept’ the ecosystem transformation brought about by multispecies invasion, while simultaneously adapting to reduce the negative impacts of the transformed community on biodiversity and people. The approach provides an independent, yet consistent, perspective to the multidimensional framework that encapsulates variation in the state of a mixed-species community (figure 1). It also parallels the typology based on abiotic conditions and biotic composition that is used to discern historical from hybrid and novel ecosystems and to assess the feasibility of restoration [77,82].

**Figure 2.**
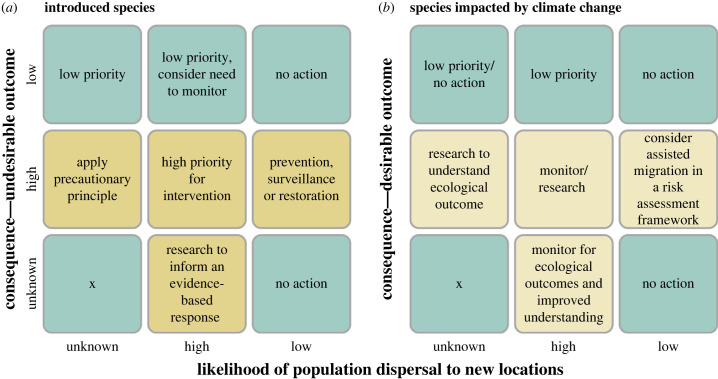
Making decisions about species movements in the face of uncertainty. The figure shows ways in which the (i) likelihood of species movement, the (ii) consequence (net positive or negative) of populations of species establishing in novel ranges, and (iii) lack of knowledge/understanding, combine to inform possible management goals for both human-mediated introductions (*a*), and natural diffusion of species populations in response to climate change (*b*) [87]. In most cases the recommendation is likely to be no action or a low priority for action (blue cells), whereas when both the likelihood and consequences of species movement are known to be significant (high), alternative responses become appropriate depending on the species involved. As outlined in the text, some species may be both introduced and moving in response to climate change, and the hard boundaries between conditions shown in the figure are not discrete, but rather represent a spectrum of contexts used as evidence to inform the decision.

The RAD categories lie on a continuum, and the iterative process of adaptive management is adopted to deal with changes in the system and conservation values [81]. In the absence of bounds between categories, the choice of R, A or D is guided by the structure and function of the mixed-species community (figure 1), conservation priorities (figure 2), scale-relevant and feasible options. For example, during the early establishment phases of invasion, when the area affected, and impacts are limited, maintaining a baseline state can be achieved by enhancing biotic resistance to invasion or eradicating alien species to prevent further spread (*resistance*, e.g. [83]). However, in cases where introduced species are widespread and interacting with native species, eradication can be damaging and disrupt desirable interactions. Instead, harnessing the novel functions of a few alien species can contribute to the functional diversity of the system (e.g. [84]). By carefully considering and integrating such nuanced approaches, mixed-species communities may be ‘directed’ towards a desired nature-positive state. When introduced species have become pervasive and strongly influence ecological interactions across the landscape [29], intensive management may be needed, from local eradications to the restoration of disrupted interactions and damaged ecosystem functions [77]. However, the outcomes of these intensive interventions are uncertain, and they may also have negative outcomes for native biodiversity and ecosystems [85]. Therefore, *accepting* novel functions provided by the transformed community can in some instances contribute positively to maintaining services threatened by the loss of native ecosystem services [86]. As the process of invasion and its consequences varies within landscapes over time, decisions to either resist, direct or accept community change are thus aimed at cumulatively increasing the net space for nature, ecosystem resilience and function.

This thinking is consistent with the concepts and standards set for ecosystem restoration, and several of the eight principles of ecological restoration are relevant—engaging stakeholders, drawing on multiple knowledge systems, supporting the recovery of ecological processes, and the value of managing ecological communities at large scales [77]. However, in a restoration context, alien species are treated as separate from native ecosystems. The presence of any alien species is considered a form of degradation, and the control of IAS is a key component of restoration activities. Restoration recognizes the occasional positive contributions of alien populations, such as nurse plants facilitating the recovery of native species [77]. Situations where alien species are considered part of the ecological community fall outside of the scope of restoration but within the domain of rehabilitation, particularly where the recovery of ecosystem function is a primary goal. Our focus in this perspective is to better understand the dynamics of mixed species communities for the purpose of achieving both biodiversity and function goals (therefore most consistent with the goals of ecological rehabilitation), given that multiple alien species are increasingly persistent members of the landscape.

### (b) Managing species movements

As mixed communities continue to emerge globally, revisiting contextual definitions of introduced species (i.e. binary alien/native, harmful/not harmful) is becoming more complex but more important than ever to inform management actions [87]. Focus on the role of dispersal in invasion biology has to date been on the human-mediated pathways and vectors responsible for transporting alien propagules from one place to the next, with the intention of managing these to prevent the introduction and spread of IAS [88]. In a NPF-BI context, the focus shifts to (i) how to manipulate the movement of propagules, individuals and populations (prevent or facilitate) to best achieve a net positive outcome for biodiversity and ecosystems, and (ii) an appreciation of the fact that both climate change and human-mediated introduction and spread of populations are driving changes in species distributions [89–91].

Multiple pieces of evidence jointly factor into decisions on if, and if so how, to intervene when species move beyond their known historic boundaries (figure 2). First is the nature of the agency involved (intentional or accidental human intervention, or a species undergoing range expansion owing to climate warming). The native range boundaries and agency involved are likely to become ever more uncertain as climates warm with the resulting re-organization of local communities [92,93]. Second, are dispersal events likely to have desirable or undesirable outcomes (figure 2)? Individual species may have both beneficial and detrimental consequences in different places or on different aspects of society or the environment [15]. Third, in which direction (negative or positive) and how large the outcomes are likely to be for those species whose effects are known? The stronger the weight of evidence for the spatial extent of the impact, the irreversibility of the change and the cost associated with it—monetary or otherwise—factor strongly into decisions on whether to intervene in population movements [94]. Finally, already well-established and widespread populations can be logistically and economically infeasible to manage. The practicality of containing, slowing or enhancing the spread and impact of species is a key consideration. The appropriate suite of responses is thus considered to enable, tolerate or suppress the multitude of species populations that are on the move for different reasons, to different extents and with different consequences for biodiversity, ecosystems and human well-being.

## 5. Conclusion

Achieving a nature-positive future given the realities of biological invasions rests on the key message from invasion biology that (i) landscapes and local communities will be host to multiple, persistent introduced species, (ii) that populations of these species will vary from place to place and over time, in the degree to which they impact natural and societal values, and (iii) that effective environmental stewardship necessarily involves integrated management of invasions by manipulating ecological processes alongside multiple, interacting drivers of change. Broad consultation and collaboration among the diverse set of actors and stakeholders involved in each situation is the cornerstone of successful environmental stewardship [30,77,95], and will be central to a NPF-BI.

While the arguments for and against the desirability of introduced species has been controversial [96], reality dictates a much more nuanced view of individual landscape stewardship into the future if we are to reach the ambitious targets set by the Kunming-Montreal Global Biodiversity Framework [97]. There is no doubt that prevention of species introductions remains an appropriate and necessary approach, whereas in other situations there is little choice but to manage and adapt to irreversible invasions. Most challenging and demanding of future research is the situation where mixed-species communities must be managed to maintain biodiversity and ecosystem function into the future. Despite the overwhelming context dependence of single and multiple species invasion outcomes, all biodiversity patterns and processes operate within physical and ecological bounds, and the variety of potential states and outcomes is finite [98]. Getting to better grips with these bounds and the distribution of states and trajectories within mixed-species communities and landscapes can overcome the view that the scale and context dependence of invasion is overwhelming.

Questions to answer in order to support this evolution in thinking include: where do mixed species communities lie in the phase space of potential community outcomes (figure 1)? What are the trajectories taken by communities within this space over time, how do ecosystem functions vary within this space, what do the trajectories of emergent function look like as communities transition, what does ‘net positive’ mean operationally at the spatial scales at which landscapes are managed, what are appropriate and achievable goals for mixed species communities, and how do management priorities, goals and approaches relate to these alternative states and ecological outcomes?

## Data Availability

This article has no additional data.
